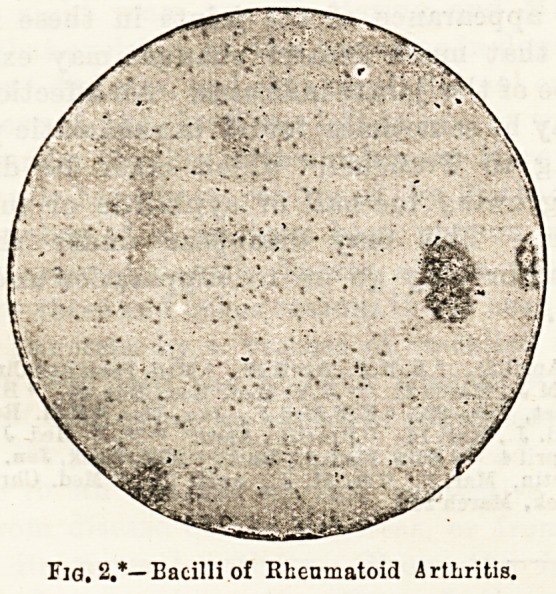# Progress in Medicine

**Published:** 1896-06-06

**Authors:** 


### Progress in Medicine.

### GOUT AND RHEUMATISM.

**Rheumatoid Arthritis.**—The pathology of this disease has been brought to the front by the researches of Wolhmann and Bannatyne,[1] which render highly probable a microbial origin for this disease. They found in twenty-four cases out of twenty-five one and the same organism often in great numbers, which probably enters the system through some catarrh of the digestive or genito-urinary organs, notably through the tonsils, and affects several joints. Here it multiplies in the synovial membrane, ligaments, cartilages and bone marrow giving rise to erosion. It secretes a poison which acts on the nervous system, producing muscular atrophy, pigmentation, and tachycardia. They obtained the organism by aspirating with aseptic precautions the affected joints. Blaxall, who worked with them, recommends that a thin film of the fluid should be dried and fixed by passing several times through the flame. The glass is then dipped in dilute acetic acid, well washed and again dried, placed on a saucer of aniline methylene blue, and left here in a dark moist chamber for three to five days. It is then washed for some hours, rinsed in distilled water, dried and mounted. Carbol fuchsine, diluted with one-third of distilled water, will give fair results in twenty-four hours, and requires a final washing in 30 per cent of alcohol. The organism appears to be a bacillus which stains only at the two ends, and resembles at first sight a diplococcus. No chains or masses are to be seen, and under Gram's method it is decolourized. Rapid staining can be effected by mixing the synovial fluid with methylene blue stain, drying and fixing and then simply washing with water. Cultivations in beef broth show after the third day gold-dust-like particles, and on agar tubes a fine film is formed by the growth. The organism has also been obtained from the blood, and does not appear to be present in joints affected with other diseases. Injections into rabbits seemed to produce some affection of the joints, but further observations are needed on this point. The disease, according to these authors, occurs chiefly in women, most often in

Fig. 1.*—Hand showing characteristic features of Rheumatoid Arthritis.

early adult life and at the climacteric, often following acute rheumatism and uterine disorders. It usually causes painful spindle-shaped swellings of the proximal interphalangeal joints of the ring and middle fingers on both hands together. Then follow in regular order affections of the wrists, the rest of the fingers, the cervical vertebræ, and the jaw, knees,

Fig. 2.*—Bacilli of Rheumatoid Arthritis.

elbows, ankles, and shoulder. There is at first no grating, but secondary osteoarthritis may follow with erosion of cartilage, eburnation, and overgrowth of bone. The temperature is raised a degree or more in the evening. Besides the anæmia, muscular atrophy, and pigmentation, we may find fingers which "go dead," and even gangrene and ecchymoses occurs. Tonic treatment with hot baths, and guiacol to the affected joints, seems to give the best results.

S. Hyde[2] has been treating the disease with a glycerine extract of healthy synovial membrane and cartilage in doses of 15 to 30 minims two or three times daily with encouraging results. The extract is made by Willows and Butler, High Holborn.

Freeman and W. Armstrong, following Ord, hold the view that the origin of the disease is intimately connected with uterine disorders, which is not irreconcilable with the bacterial discoveries abovementioned. Thus Armstrong[3] mentions that out of 146 female cases 120 suffered from uterine or ovarian troubles, which almost always preceded the joint lesions. He recommends first treatment of the uterine lesions, and then electric baths, with either the constant or the alternating current to allay the spinal irritability which he thinks is the cause of the joint lesions. Thus he gives six galvanic baths on successive days, followed by alternate galvanic and mineral water baths, with tonic treatment and massage. Of course, on Bannatyne's theory, the uterine trouble merely affords entrance to the germs, and the spinal and nerve lesions are caused by the toxins formed in the joints. The late Dr. Brabazon[4] left an analysis of 100 cases under his care up to a year ago. He believed that it was in no way connected with gout or rheumatism, occurred most often in women of a neurotic type, and depended on an affection of the trophic centres and trophic nerves, and was accompanied by profound constitutional changes, to which the sufferer finally succumbed. These changes included general emaciation, tachycardia, sweating palms, a variable temperature, and anæmia. Very pathognomonic, too, was the worn, hopeless expression of face which he noticed. He also recommended iron, arsenic, cod-liver oil, electricity, and hot baths, as well as occasionally the forcible breaking down of adhesions in the affected joints. There seems great suitability in the name he suggested of pernicious arthritis. Cantagruel[5] advises tonics and iodides internally, and arsenical baths. One or even two drachms of arseniate of soda and three or four ounces of bicarbonate of soda are used for each bath.

The symmetrical character of the joint lesions will be found not only in this disease, according to E. S. Reynolds, but also in true chronic rheumatism, when the hands are affected. He holds[6] that in both the joints of the hands are symmetrically affected, and to an equal degree on both sides, and that the deformities produced are equal and symmetrical, except when interfered with by old injuries or Dupuytren's contractions. Thus he effects a diagnosis from gout, in which the joints affected, the extent of the affection, and deformities produced are asymmetrical.

**Rheumatism.**—Churton[7] recently analysed 135 cases with the result that he believes that chills only aid the attack of micro-organisms, and are not the sole cause of the disease, and that chorea is a form of rheumatism. Another symptom in childhood is often an affection of the cervical vertebræ, sometimes leading to anchylosis. This to be distinguished from simple torticollis and caries. Haig,[8] again, argues that uric

* We are enabled to reproduce these figures from the forthcoming work on Rheumatoid Arthritis, by the courtesy of the author, Dr. Bannatyne.

acid retention is the cause of rheumatism, and that drugs are useful just in proportion to their power of eliminating it. Heat dissolves it in the blood and cold precipitates it in the joints. Beverley Robinson, accepting the miasmatic theory as the best brought forward, regards the excess of lactic acid as a consequence, not a cause, of the disease, and thinks the addition of an alkali to other medicine desirable for its elimination. He objects to excessive doses of salicylates, and finds better results from combining with it a little colchicum. With great success, too, he has employed acetate of potash and chloride of ammonium in ten or fifteen grain doses of each every two hours. L. F. Bishop,[9] in summing up the evidence for the various theories, confesses that it is on the whole in favour of a miasmatic origin, and one rather like that of malaria than a bacterium. Though the production of endocarditis by Richardson's injection of lactic acid, and the appearance of rheumatism after the administration of lactic acid in diabetes have some weight, he would regard the excess of lactic rather as the secretion of some microbe. Probably several diseases are included under the same designation. If alkalies are given for the sake of preventing heart complications he would order drachm doses of bicarbonate of soda often enough to render the urine alkaline. Salophen (which has been found very serviceable in sciatica and similar affections by the writer) is recommended by H. S. Pearse[10] for acute rheumatism. It has no irritating effect on the stomach, being only decomposed in the intestine. He found the fever disappeared more quickly than under salicylates, and that there were few or no renal or cerebral complications or depression from its use.

Bourget for two years has treated all acute rheumatic cases by external applications only. He employs an ointment to the joints without friction, consisting of salicylic acid and turpentine, of each half an ounce, lanolin and lard, of each four ounces. This caused a rapid fall of temperature and pain without the accidents which occur with internal medication.[11] Salacetol is also recommended by an Italian author, and saligenin, the active principle of salicin, by Lederer.[12] He claims that its action is rapid and certain, and without the unpleasant consequences of salicylic acid. It may be given as a powder, in doses of seven to fifteeen grains every two hours. Linossier and Lannois find that[13] salicylate of methyl is readily absorbed by the skin. The liquid is painted on a limb and covered by oiled silk and cotton wool. Sixty grains applied in this manner will cause the excretion of twenty grains of salicylic acid in the urine in twenty-four hours, without disturbing the digestion or irritating the skin.

**Gout.**—The important researches of Klemperer on the uric acid question have recently been reported. He denied that the blood in gout is near saturation, or that it has more than is found in other diseases, such as leucæmia. Haig and others give salicylates to increase the excretion of uric acid, and Haig claims to have increased the excretion thirteenfold in persons of rheumatic habit; but as Klemperer and Von Jaksch find little or none in the blood of such persons, this increase must be due to increased production. Bohland[14] seems to have proved this on healthy men, for while the uric acid excreted was nearly doubled, so too was the number of the leucocytes, from which probably the acid is formed. We may add that Neusser noted that in gout an excess of leucocytes was present in the blood. Thus Lockhart Gillespie concludes that the evidence tends to show that gout depends on deficient oxidation of the nucleo compounds or on an excess of their products. As to treatment, Jaccoud[15] in the acute attack gives nothing for the first five days except a very mild diuretic and anodynes to the joints. If it is obstinate he then orders hydrobromate of quinine with digitalis, or if the pains are increasing full doses of salicylates, provided the kidneys are healthy. In sub-acute attacks he adds colchicum seeds to the quinine and digitalis. In visceral complications coming on after years of typical gout colchicum is given, and the joints usually affected are blistered.

Klemperer[16] notices that urea is a powerful solvent of uric acid as well as a diuretic, and prefers it to piperazin. He gives ten-grain doses every hour for gravel, and after the first day or two a single dose of twenty grains daily. Rœntgen's rays have been employed for the diagnosis of gouty and rheumatic joint disease, and to determine the amount of change in the bones as a guide to the probable value of local treatment. Thus, Huber[17] determined the absence of bone changes in chronic rheumatism in the case of a woman, and noticed the transparency of uric acid deposits compared with true osseous outgrowths. A beautiful series of engravings of the various changes, erosions, atrophy, and dislocations of the bones, is given by E. Reynolds,[18] with photographs of the external appearance of the joints in these diseases, showing that much greater changes may exist than the shape of the joints indicates. An affection of the nails may be sometimes found in rheumatic patients, according to Tronchet,[19] which must be diagnosed from ingrowing toe-nail or syphilitic or tubercular onychnia. This may disappear under rheumatic treatment, or may go on to supparation and require avulsion.

1 Lanc., April 25. 2 B.M.J., April 18. 3 Med. Press and Circ., Dec. 25, 1895. 4 B.M.J., March 21. 5 N.Y. Med. Rec., Dec. 28. 6 B.M.J., Feb. 15. 7 Lancet, Feb. 22. 8 N.Y. Med J., Jan. 11. 9 Med. Rec., Feb. 1. 10 N.Y. Med. J., Mar. 14. 11 Practit, April. 12 N. Y. Med J., April 11. 13 Lanc., April 4. 14 Edin. Med. J., Mar. 15 Med. Week, Jan. 15. 16 Am. M.S. Bulletin, Mar. 7. 17 B. M. J., Aprll 25. 18 Med. Chron., April. 19 Med. Week, March 20.

## Figures and Tables

**Fig. 1. f1:**
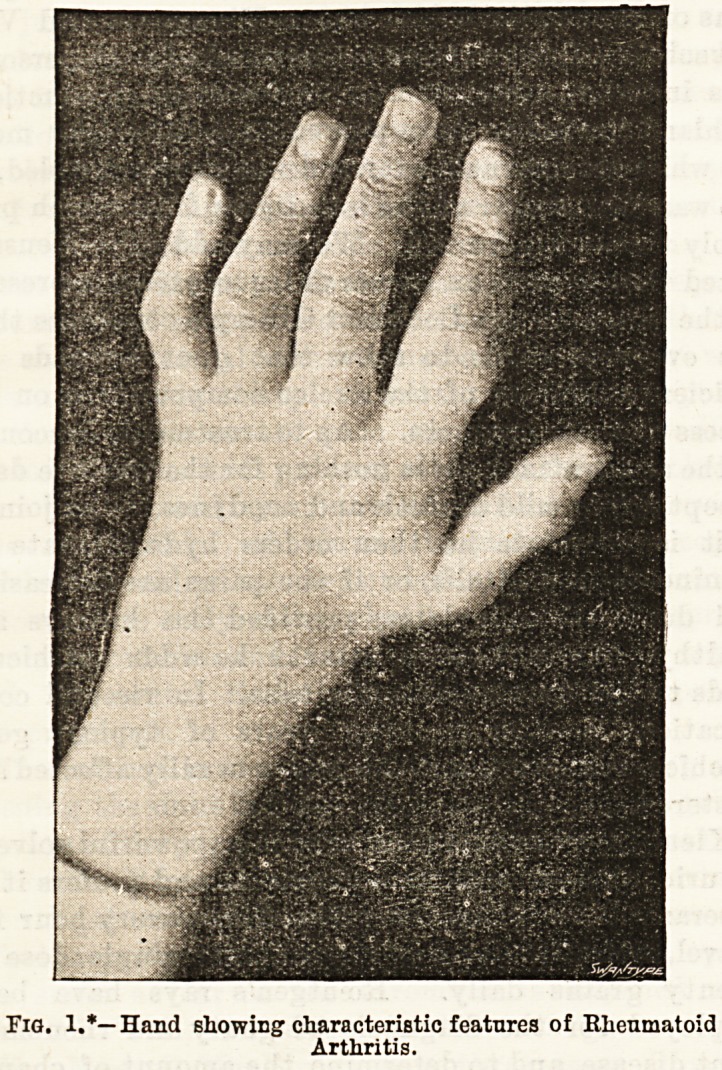


**Fig. 2. f2:**